# An Eye-Opening Approach: Cancer of Unknown Primary Source With Choroidal Metastasis Case Report

**DOI:** 10.2196/77895

**Published:** 2026-02-05

**Authors:** Erin Kaser, Vedin Barve, Alexander Blaschke, Constance Fry, Corey Waldman, Sarah Hackman, William Kelly

**Affiliations:** 1 Department of Internal Medicine, The University of Texas Health Science Center at San Antonio, 7979 Wurzbach Road, San Antonio, TX, 8232, United States, 1 210-450-1000; 2 Long School of Medicine, The University of Texas Health Science Center at San Antonio, San Antonio, TX, United States; 3 Division of Podiatry, Department of Orthopaedics, The University of Texas Health Science Center at San Antonio, San Antonio, TX, United States; 4 Department of Opthalmology, University of Tennessee Health Science Center, Memphis, TN, United States; 5 Department of Opthalmology, The University of Texas Health Science Center at San Antonio, San Antonio, TX, United States; 6 The University of Texas Health Science Center at San Antonio, San Antonio, TX, United States

**Keywords:** choroidal, metastasis, non-small cell lung cancer, cancer of unknown primary origin, multi-modal therapy

## Abstract

Choroidal metastases (CM) represent a rare but clinically significant manifestation of systemic malignancy, most frequently from lung cancer.

The choroid’s vascular anatomy allows hematogenous tumor seeding. Although CM may be the first clinical sign of an underlying malignancy, evidence guiding its management in the modern immunotherapy era remains limited, as most published cases predate the widespread use of immune checkpoint-inhibitors.

We describe a 33-year-old male patient presenting with ocular pain and visual disturbance, who was found to have an amelanotic choroidal lesion. Systemic workup revealed small pulmonary nodules and an iliac crest lesion. Sequential biopsies suggested that this was metastatic adenocarcinoma of unknown primary origin, but most likely of lung origin, without actionable mutations or PD-L1 (programmed death-ligand 1) expression. Management required multidisciplinary coordination and included carboplatin, paclitaxel, and pembrolizumab, followed by radiation to the orbit, iliac crest, and mediastinal sites of disease. Unfortunately, he experienced progression while on maintenance immunotherapy with new rib and brain lesions, for which he underwent treatment with platinum, pemetrexed, and bevacizumab with additional radiotherapy. Despite loss of vision in the affected eye, he achieved durable disease control and remains free of radiographic recurrent disease>4 years after diagnosis.

This case illustrates that multimodality salvage strategies—integrating systemic therapy with aggressive local radiation—can provide unexpectedly prolonged survival even after immunotherapy failure. Importantly, current guidelines offer minimal direction on managing CM in this context, and prior case reports do not reflect present-day treatment realities. The key message for clinicians is that CM should not automatically be approached with palliative intent; carefully selected patients may benefit from an oligometastatic strategy that actively targets limited metastatic sites to prolong survival. Our findings underscore the need for ophthalmology, radiation oncology, and medical oncology collaboration when vision-threatening or occult metastatic lesions arise.

For readers, the takeaway is that choroidal metastasis—particularly in the era of immunotherapy—warrants individualized, multidisciplinary evaluation rather than default palliation. Our case demonstrates that coordinated multimodality management can achieve long-term disease control, highlighting a treatment paradigm worth considering for selected patients and calling for updated guidelines that reflect modern therapeutic capabilities.

## Introduction

Rare presentations of cancer can include metastases to the orbit through hematogenous dissemination. The uveal tract is a highly vascular complex made from the choroid, ciliary body, and iris. Importantly, the eye contains the blood-retinal barrier, analogous to the blood-brain barrier. However, the choroid layer lies outside retinal pigment epithelial tight junctions and is therefore exposed to unimpeded drug delivery from the vasculature [1]. One of the most common sources of choroidal metastases is primary lung cancer, with 88% of lung metastases to the eye involving the choroid, with an associated overall survival at one-year of 54% [2]. Consequently, choroidal metastases (CM) of unknown origin are often treated similarly to lung cancer as the clinical, imaging, and histopathologic features of choroidal lesions frequently resemble those seen in lung cancer patients [3,4]. The prognosis for carcinoma of unknown primary (CUP) with CM is generally poor, with median overall survival ranging from 3 to 12 months, as patients tend to present with advanced and disseminated disease [5,6].

## Case Presentation

### Patient Information

The patient was a 33-year-old male at the time of initial presentation with no significant past medical history. He presented with right ophthalmalgia, blurry vision, and sinus congestion. Clinical course was as follows (Table 1).

**Table 1.** Timeline of key events in the described patient presentation

Day	Description of key events.
Day 0-2	Initial presentation: chief complaint of right ophthalmalgia and blurry vision. Ophthalmology observed an amelanotic right choroidal lesion. MRI^a^ Orbit showed enhancing plaque of the posterior-lateral wall of right globe. CT^b^ chest showed RML and RUL nodules
Day 44-113	Pathological workup: right intraophthalmic choroidal biopsy with scant atypical cells. PET (Positron Emission Tomography) revealed non-avid RML^c^ and RUL^d^ nodules and mildly avid L iliac crest. R choroidal FNA^e^ read as metastatic carcinoma, compatible with pulmonary adenocarcinoma. L iliac biopsy with further suggestion of lung origin (CK7, TTF1+)
Day 125-313	Initial chemoimmunotherapy: started on carboplatin, paclitaxel and pembrolizumab for 4 cycles followed by pembrolizumab maintenance. Completed stereotactic radiation to R orbit
Day 293-324	Disease progression: clinical and radiographic progression at ribs and lungs as well as intracranial site
Day 313-743	Oligometastatic salvage therapy: given radiation to chest wall and left frontal operculum. Started on carboplatin, pemetrexed, and bevacizumab for 4 cycles followed by pemetrexed and bevacizumab maintenance. Bevacizumab was continued as maintenance for 14 cycles
Day 743-Present	Surveillance: no clinical or radiographic evidence of disease progression as of day 1563.

a MRI: magnetic resonance imaging.

b CT: computed tomography.

c RML: right middle lobe.

d RUL: right upper lobe.

e FNA: fine-needle aspiration.

## Ethical Considerations

The patient involved in this case report has given his informed consent authorizing the use and disclosure of his health information.

## Clinical Findings

**Figure 1.**
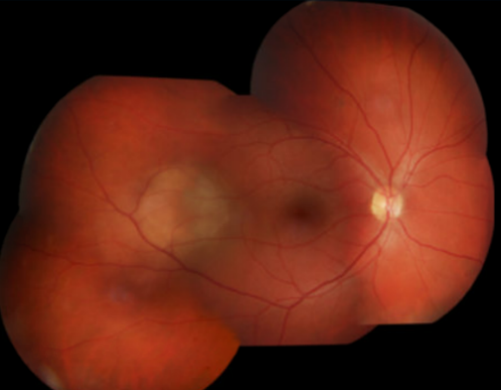
Ophthalmologic evaluation showed an amelanotic right choroidal lesion.

Evaluation by ophthalmology revealed an amelanotic right choroidal lesion (1.04 × 1.15×.31 cm, Figure 1). The patient had 20/20‐1 vision bilaterally with full visual fields.

## Diagnostic Assessment

**Figure 2.**
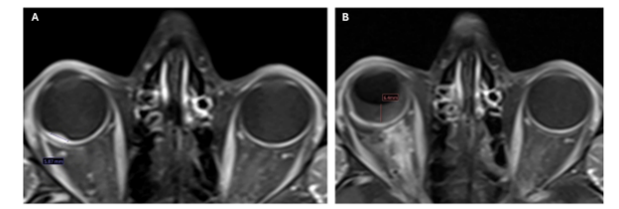
(A) MRI orbit (Day 0) showed enhancing plaque along the posterior-lateral wall of his right globe. (B) MRI orbit (Day 1,123) showed new right optic nerve enhancement.

**Figure 3.**
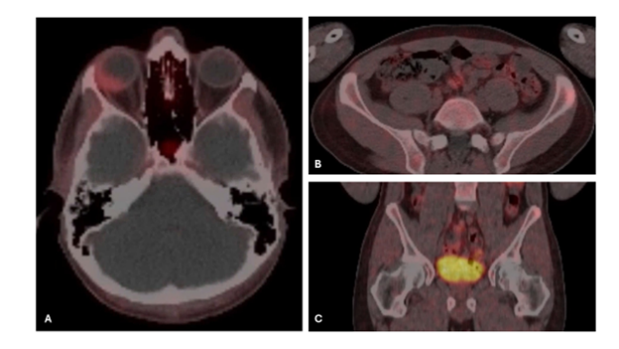
DOTATATE PET Imaging on d 64 demonstrating sites of metastatic involvement(A) Axial DOTATATE PET of the orbits showing mild radiotracer avidity within the right choroidal lesion (SUV 2.6; Krenning score 1), corresponding to the enhancing plaque previously identified on MRI; (B) Axial PET of the pelvis showing a mildly avid lytic lesion in the left iliac crest (approximately 1 cm), consistent with metastatic involvement; (C) Coronal PET confirming the left iliac crest lesion with focal uptake.

**Figure 4.**
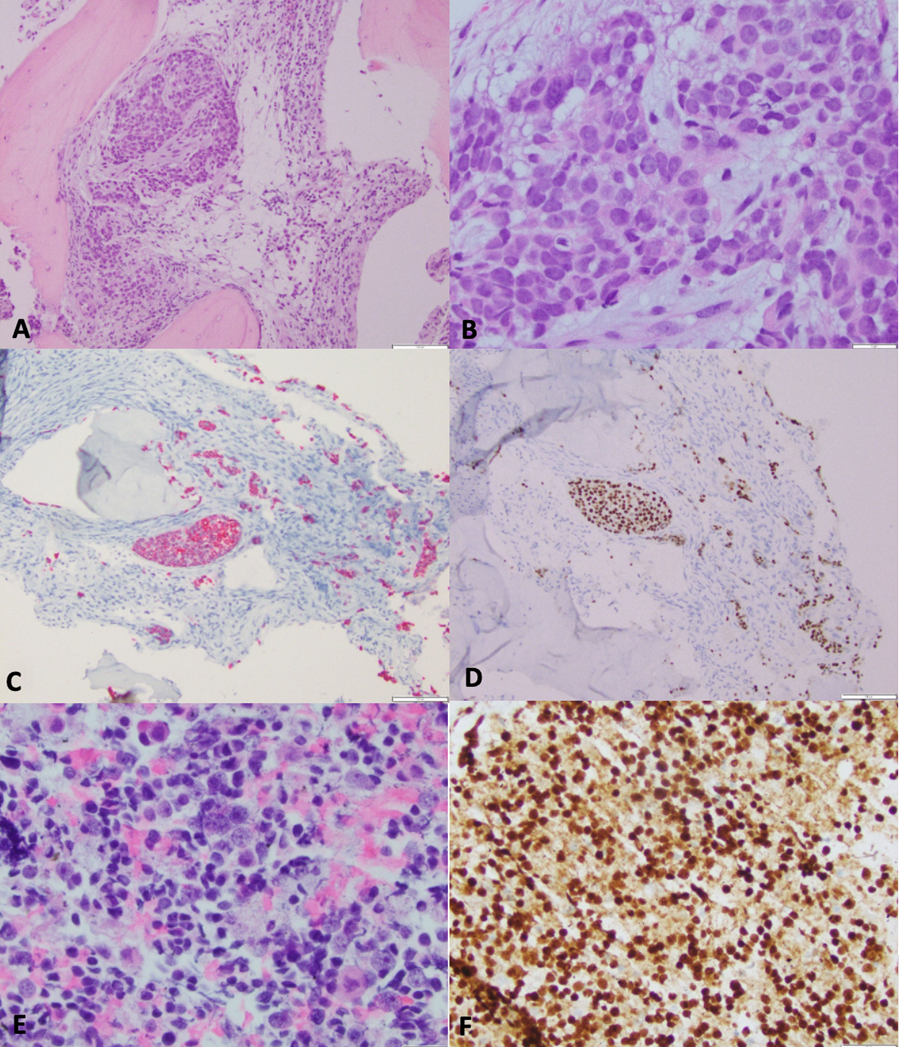
Iliac crest biopsy (Day 113) and pulmonary lymph node biopsy (Day 114). (A, B) Iliac crest bone lesion, H&E, 10 x and 40 x: tumor cells with bone; (C) Iliac crest bone lesion, Cytokeratin AE1/3 immunostaining, 10 x: cytokeratin stain confirming the tumor cells are carcinoma; (D) Iliac crest bone lesion, TTF-1 immunostaining, 10 x: TTF-1 stain supports lung origin; (E) Pulmonary lymph node: H&E, 40 x; tumor cells are morphologically comptabile with those in bone; (F) pulmonary lymph node: TTF-1 immunostaining, 20 x: TTF-1 stain supports lung origin. TTF: thyroid transcription factor.

MRI Orbit on Day 0 (Figure 2A) confirmed an enhancing plaque along the posterior-lateral wall of his right globe. A CT Chest (Day 2) demonstrated a right middle lobe (RML) (0.5cm) and right upper lobe (RUL) nodule (0.2cm). The patient underwent intraophthalmic choroidal biopsy (Day 44). Pathology read as scant atypical cells, with few cells showing ‘neuroendocrine-like nuclear features.’ A DOTATATE PET (Positron Emission Tomography) on Day 64 was performed, which showed mild avidity in the right globe mass (SUV 2.6, Krenning 1) (Figure 3A), a nonavid RML nodule (0.5cm, below PET resolution) and RUL nodule (0.2cm), and a mildly avid left iliac crest lesion (1cm) (Figure 3B, C). The patient subsequently underwent a right choroidal fine-needle aspiration (FNA) on Day 69, with pathology read as metastatic carcinoma, compatible with pulmonary adenocarcinoma. Notably, the histology did not resemble a retinal pigment epithelium tumor and was positive for CK7 and TTF1 but negative for PD-L1, thyroglobulin, PAX8, CD20, GATA3, CD3, and Melan-A. CK20 was only weakly focally positive. Synaptophysin highlighted pieces of retina but not the tumor. Molecular testing showed no actionable mutation in EGFR, KRAS, BRAF, or HER2. Unfortunately, testing for ALK, RET, ROS1, NTRK1/3 and MET was inconclusive due to insufficient sample. A left iliac biopsy (Day 113, Figure 4) and mediastinal lymph node (4R) FNA biopsy (Day 114) were also read as carcinoma showing CK7 and TTF1 positivity. An insufficient sample of tumor was procured for sequencing; however, immunohistochemical (IHC) analysis was negative for EGFR, ALK, ROS, and BRAF driver mutations. No PD-L1 expression (0%) was observed. The patient was discussed in a multidisciplinary tumor board with the ultimate determination that this was metastatic adenocarcinoma of unknown primary, but most likely of pulmonary origin and to treat as non-small cell lung cancer (NSCLC).

## Therapeutic Intervention

Following tumor board discussion, treatment was initiated with carboplatin (AUC 6), paclitaxel (200mg/m^2^), and pembrolizumab (200mg) every 21 days (C1D1 on Day 125) for 4 cycles, with pembrolizumab continued as maintenance after cycle 4. Alternative therapies considered included a palliative approach with chemotherapy alone, but age, functional status, limited extent of disease, and the patient’s wishes to pursue an aggressive therapeutic approach led to the chosen treatment protocol. Paclitaxel was chosen over pemetrexed as the taxane used because it was deemed to cover more histologies in the event that the cancer was not actually of bronchopulmonary origin. Patient completed stereotactic radiosurgery (SRS) (Day 173) with 36 Gray (Gy) in 4 fractions (fx) to the right orbit lesion. Following cycle 4, he was placed on pembrolizumab maintenance. PET (Day 211) showed an interval increase in L iliac crest lesion and increased conspicuity of R lung base grouped nodularity, but otherwise stable R paratracheal (0.7cm) and mediastinal (0.4cm) lymph nodes and a decreased RML (0.3cm) pulmonary nodule. He also completed radiation to the left iliac (Day 252, 50Gy in 5 fx), RML (Day 252, 50Gy in 5 fx), and mediastinal nodes (Day 273, 40Gy in 10 fx). However, he began having rib pain and PET/CT (Day 293) showed a new lesion in the left 4th rib and RLL, as well as an increase in additional right pulmonary nodules, concerning for progression. Concurrently, MRI Brain (Day 324) had also shown a new left frontal gyrus lesion. Given his age and continued limited disease involvement, an oligometastatic approach was used, and the patient completed radiation to the left chest wall (Day 313, 50Gy in 5 fx) and left frontal operculum (Day 337, 1 fx). Pembrolizumab was stopped (last dose on Day 315) due to radiographic progression, and he was started (C1D1 on Day 344) on carboplatin (AUC 5), pemetrexed (500mg/m^2^), and bevacizumab (15mg/kg) every 21 days for 4 cycles, with pemetrexed and bevacizumab continued as maintenance after cycle 4. Pemetrexed and bevacizumab were held after 14 cycles (last dose on Day 743) in preparation for cataract removal of the right eye, and he was subsequently placed on surveillance with MRI Brain and PET imaging every 3 months.

## Follow-Up and Outcomes

He developed eye pain, and MRI orbit (Day 1123, Figure 2B) showed right optic nerve enhancement, which was new from previous imaging and felt secondary to prior radiotherapy. He received one dose of intraocular bevacizumab injection, which completely resolved the pain. The patient had developed blindness in his right eye after initial treatment, but remained with 20/20 vision on the left. He maintained his employment and continues with regular follow-up and surveillance imaging every 3 months. His scans as of Day 1563 show no evidence of recurrence.

## Discussion

Given CUP with CM tends to present with late, advanced, and disseminated disease, the general treatment protocol is palliative, focusing on symptom control and vision preservation. For most patients, external beam radiotherapy is the standard local treatment for symptomatic choroidal metastases, with systemic therapy including platinum- or taxane-based chemotherapy used for widespread disease [7]. Systemic therapy for CM of CUP often mirrors lung cancer regimens because empirical chemotherapy and targeted therapies have demonstrated efficacy in controlling both systemic and ocular disease in lung cancer patients [8-10]. For metastatic non-small cell lung cancer, platinum-based chemotherapy with PD-1 blockade is the standard first-line treatment; however, data regarding CM in the age of immunotherapy are limited. Recently, Li et al presented a case of a 65-year-old male patient with NSCLC and CM who demonstrated ultrasonic regression, improved visual acuity, and symptomatic relief after treatment with pemetrexed and capecitabine plus pembrolizumab [11]. A second case report by Matsuyama et al describes treatment with nivolumab plus ipilimumab plus chemotherapy, which resulted in symptomatic improvement and no relapse at 18 months follow-up [12]. These isolated cases underscore both the rarity of CM and the need for evidence-guided management strategies.

Bevacizumab, an extracellular VEGF-A inhibitor, has strong anti-angiogenic properties and has been used to treat CM from pulmonary, breast, and colorectal metastases. For instance, Riess JW et al described a case series of 3 patients with NSCLC and CM who were treated with a backbone of platinum-based chemotherapy plus bevacizumab [13]. The first patient received SRS to the symptomatic CM with improved vision and 12 months of progression-free survival. The second patient was found to have CM after right eye vision loss. This vision normalized following whole-brain radiotherapy that included the choroidal regions. Thereafter, the patient received 14 cycles of chemotherapy followed by erlotinib for a discovered EGFR mutation and progression. The third patient tolerated 16 months of chemotherapy followed by a recurrence of CM, which was treated with an ALK inhibitor for a found translocation. Furthermore, George et al reported on a 42-year-old female patient diagnosed with NSCLC with CM who achieved complete response of her 1.2cm CM following 3 cycles of carboplatin, gemcitabine, and bevacizumab, and was still alive with reported normal vision following her 7th maintenance dose of bevacizumab [14].

In the case of our patient, timely referral to ophthalmology led to recognition of a uveal lesion within three weeks of symptom onset. PET imaging can be helpful in defining dominant lesions and disease burden but histologic evaluation is necessary to guide diagnosis. In situations such as ours, where the primary site cannot be readily identified from imaging, judicious use of immunohistochemistry and molecular profiling is called for. In the era of immunotherapeutics, the median survival of newly diagnosed NSCLC without targetable mutation ranges from 14 to 22 months [15,16]. At the time of writing, our patient has survived 4 years with no radiographic or clinical signs of recurrence. While we must be careful not to read too much into an isolated case, it is the authors’ belief that an aggressive oligometastatic approach with multimodality salvage options may be best for all NSCLC patients or patients with suspected primary lung malignancy who initially present with choroid disease and limited disease elsewhere.

As presented, this case illustrates an innovative management strategy for choroidal metastasis in the era of immunotherapy. While prior reports describe short-term responses to chemotherapy, anti-VEGF therapy, or immune checkpoint inhibitors, none have documented long-term survival following an oligometastatic approach that was applied both at diagnosis and after systemic progression. Our patient’s survival of more than 4 years exceeds historical expectations for metastatic NSCLC without targetable alterations or PD-1 expression and highlights the potential benefits of aggressive local therapy even at the cost of vision.

The substantial collaboration required to execute this treatment approach is equally important. Coordination between imaging, multiple ophthalmologic biopsies, and subspecialty pathologic review was essential for diagnosis. Treatment required communication between medical and radiation oncology to prioritize systemic control while delivering targeted radiation to multiple metastatic sites. This approach reflects the real-world practice and serves as a model for clinicians managing similarly rare and complex presentations.

## Conclusion

CM remains an uncommon presentation of systemic malignancy, and contemporary management is challenged by the absence of evidence-based guidelines that reflect the current era of immunotherapy. Our case demonstrates that even after progression on checkpoint inhibitors, carefully selected patients may achieve prolonged survival through a coordinated multimodality approach that integrates systemic therapy with site-directed radiation. This experience suggests that CM should not automatically be considered a purely palliative entity, particularly when disease burden is low and lesions are anatomically accessible for local therapy.

More broadly, this report emphasizes the importance of early ophthalmologic evaluation for unexplained visual symptoms, the value of thorough systemic staging when CM is suspected, and the critical role of multidisciplinary collaboration involving medical oncology, radiation oncology, and ophthalmology. As prior published cases predate modern immunotherapy, this case highlights an unmet need for updated clinical guidance on managing CM in patients who have progressed on or failed to respond to immune checkpoint inhibitors.

## Abbreviations

ALK: anaplastic lymphoma kinase
AUC: area under the curve
BRAF: B-rapidly accelerated fibrosarcoma (BRAF) gene
CK7: Cytokeratin 7
CM: choroidal metastases
CT: computed tomography
CUP: cancer of unknown primary
CxDy: Cycle x Day y
DOTATATE PET: DOTA-Tyr³-octreotate Positron Emission Tomography
EGFR: epidermal growth factor receptor
FNA: fine-needle aspiration
Fx: fraction
Gy: gray (unit of radiation dose)
IHC: immunohistochemistry
MRI: magnetic resonance imaging
NSCLC: non-small cell lung cancer
PAX8: paired box gene 8
PD-L1: programmed death-ligand 1
RLL: right lower lobe
RML: right middle lobe
ROS1: ROS proto-oncogene 1 receptor tyrosine kinase
RUL: right upper lobe
SRS: stereotactic radiation
TTF1: thyroid transcription factor-1
VEGF-A: vascular endothelial growth factor – A
